# Lymphocyte-to-C reactive protein ratio as novel inflammatory marker for predicting outcomes in hemodialysis patients: A multicenter observational study

**DOI:** 10.3389/fimmu.2023.1101222

**Published:** 2023-03-02

**Authors:** Xinpan Chen, Wang Guo, Zongli Diao, Hongdong Huang, Wenhu Liu

**Affiliations:** Department of Nephrology, Beijing Friendship Hospital, Capital Medical University, Beijing, China

**Keywords:** inflammation, hemodialysis, lymphocyte-to-C reactive protein ratio, prognosis, biomarker

## Abstract

**Background:**

Patients undergoing hemodialysis experience inflammation, which is associated with a higher risk of mortality. The lymphocyte-to-C reactive protein ratio (LCR) is a novel marker of inflammation that has been shown to predict mortality in patients with malignant cancer. However, the utility of LCR has not been evaluated in patients undergoing hemodialysis.

**Methods:**

We performed a multi-center cohort study of 3,856 patients who underwent hemodialysis as part of the Beijing Hemodialysis Quality Control and Improvement Project between 1 January 2012 and December 2019. The relationship between LCR and all-cause mortality was assessed using a restricted cubic spline model and a multivariate Cox regression model. An outcome-oriented method was used to determine the most appropriate cut-off value of LCR. Subgroup analysis was also performed to evaluate the relationships of LCR with key parameters.

**Results:**

Of the 3,856 enrolled patients, 1,581 (41%) were female, and their median age was 62 (53, 73) years. Over a median follow-up period of 75.1 months, 1,129 deaths occurred. The mortality rate for the patients after 60 months was 38.1% (95% confidence interval (CI) 36%–40.1%), resulting in a rate of 93.41 events per 1,000 patient-years. LCR showed an L-shaped dose-response relationship with all-cause mortality. The optimal cut-off point for LCR as a predictor of mortality in hemodialysis patients was 1513.1. An LCR of ≥1513.1 could independently predict mortality (hazard ratio 0.75, 95% CI 0.66–0.85, P<0.001).

**Conclusions:**

Baseline LCR was found to be an independent prognostic biomarker in patients undergoing hemodialysis. Implying that it should be a useful means of improving patient prognosis and judging the timing of appropriate interventions in routine clinical practice.

## Introduction

Persistent inflammation has been shown to facilitate the development of various diseases and increase the associated mortality rates, including for chronic kidney disease, chronic obstructive pulmonary disease, cardiovascular disease, and diabetes (1–5). For patients with end-stage renal disease who are undergoing hemodialysis, the complications of anemia, malnutrition, and vascular calcification are considered to increase morbidity and mortality (6–8), and the underlying mechanisms are inextricably linked to the persistent inflammatory state (9, 10). This persistent inflammatory state can also lead to protein-energy wasting, resulting in malnutrition, which is associated with a high risk of mortality in patients undergoing hemodialysis (11, 12). Therefore, paying close attention to the inflammatory status of such patients is important.

Although markers such as PCT, IL-6, TNF-α, and MMP-9 have been shown to be sensitive and accurate means of predicting inflammation and mortality, such assays are either expensive or unavailable in clinical laboratories. Therefore, an alternative prognostic marker that is simple, inexpensive, and convenient to measure, and that is available in most dialysis laboratories, is still required. Complete blood counts are easy and commonly performed measurements that can help predict inflammation, and several combinations of hematological indices, such as the neutrophil-to-lymphocyte ratio (NLR) and the platelet-to-lymphocyte ratio (PLR), have been developed for use as prognostic markers in patients undergoing hemodialysis (13–15). In addition, other makers of systemic inflammation, such as the prognostic nutritional index (PNI) and the Glasgow prognostic score (GPS), can be used to predict outcomes in such patients by quantifying their nutritional and immunological statuses (16, 17).

Recently, the lymphocyte-to-C reactive protein ratio (LCR), which can be easily calculated using the lymphocyte count and serum CRP concentration, has been shown to be associated with the severity of inflammation in and the mortality of patients with malignant disease. Specifically, low LCR has been shown to be an independent predictor of overall survival in patients with colorectal cancer, gastric cancer, hepatocellular carcinoma, bladder cancer, or rectal cancer (18–23). Thus, LCR has shown potential as a predictor of inflammation and mortality. However, to date, no studies have evaluated the relationship between LCR and mortality in patients undergoing hemodialysis. We hypothesized that LCR may also be a useful predictor of mortality in these patients, and in the present study, we performed pooled analyses of clinically relevant variables to determine whether LCR is associated with the overall survival of patients undergoing hemodialysis and whether it interacts with other clinical variables.

## Methods

### Participants and study design

We performed a retrospective multi-center cohort study of individuals selected from the 6,126 participants in the Beijing Hemodialysis Quality Control and Improvement Project, during which data was collected at 138 dialysis centers between 1 January 2012 and 31 December 2019. The inclusion criteria were age ≥ 18 years and hemodialysis three times a week for at least 3 months. The exclusion criteria were as follows (1): duration of hemodialysis < 3 months; (2) previous treatment by peritoneal dialysis; (3) organ transplantation; (4) malignant disease; (5) autoimmune disease, or chronic or acute infectious disease; and (6) missing baseline data. The existence of acute or chronic infection was determined based on the admission diagnosis of patients that explicitly stated infection (for example, pneumonia) or that provided evidence of infection (detected bacteria or virus). After the application of these criteria, 3,856 patients were eligible for enrolment in the present study (**Figure S1**). The study was performed in accordance with the principles of the Declaration of Helsinki and was approved by the Human Ethics Committee of Beijing Friendship Hospital.

### Clinical data

Variables and potential confounders were selected for study by considering clinical guidelines and the results of previously published studies. Only data collected during the first assessment made after the initiation of hemodialysis were included. The baseline demographic data (age and sex) and biochemical/hematological data (hemoglobin, albumin, platelet count, neutrophil count, lymphocyte count, C-reactive protein, creatinine, urea, calcium, parathormone, phosphorus, Fe, ferritin and UIBC) were obtained within the first month of hemodialysis. The biochemical/hematological data were standardized before being recorded in the Beijing Hemodialysis Quality Control and Improvement Project database to minimize variability in the measurements made by the various dialysis laboratories. All of these data were collected from the project database. The patients were followed from the initiation of hemodialysis until they were transferred to another hemodialysis center, they were changed to peritoneal dialysis, they underwent kidney transplantation, they were lost to follow-up, they died, or the end of the study period on 31 December 2019. The LCR was calculated as lymphocyte count (10^9^/L)/C-reactive protein (mg/L) × 10^4^. For the subsequent stratified analyses, on the basis of the laboratory reference ranges and the Kidney Disease Outcome Quality Initiative (KDOQI) guidelines, the biochemical data were defined as normal or abnormal. The normal values were as follows: hemoglobin 100–130 g/L, platelet count 125–350×10^9^/L, neutrophil count 1.8–6.3×10^9^/L, albumin >35 g/L, calcium 2.1–2.52 mmol/L, parathormone 150–300 pg/μl, phosphorus 1.13–1.78 mmol/L, ferritin 200–500 ng/ml.

### Outcome

The overall survival time was defined as the interval between the initiation of hemodialysis and the date of death, transfer to another dialysis center, the change to peritoneal dialysis, kidney transplantation, withdrawal from the study, or the end of the study period (31 December 2019). We aimed to evaluate the relationship between LCR and overall survival and to identify the most appropriate cut-off value of LCR for prognostic use in patients undergoing regular hemodialysis.

### Statistical analysis

Continuous datasets with skewed distributions are presented as median (interquartile range) and were compared using the Mann-Whitney test. Categorical data are expressed as numbers (percentages) and were compared using Pearson’s χ^2^ test. Spearman correlation analysis was used to identify linear relationships between LCR and selected variables. Univariate and multivariate Cox proportional hazards regression models were used to identify the risk factors for mortality and to provide hazard ratios (HRs) for each variable. Sex and age were included in the multivariate Cox regression model and other potentially confounding variables were selected based on the results of univariate analysis (*p*<0.1 was defined as indicating a significant association with all-cause mortality). For the time-to-event analysis, survival curves were generated using the Kaplan-Meier method and compared using the log-rank test.

The non-linear relationship between LCR and HR was visualized using restricted cubic splines. The optimal cut-off value for LCR was determined using the ‘surv_cutpoint’ formula in the ‘survminer’ R package, which is an outcome-oriented method that provides a cut-off value with the closest relationship with the outcome. The participants were divided into high- and low-LCR groups based on the optimal cut-off value. Trend tests were performed by assigning a median value to each quartile of the LCR, which was then modeled as a continuous variable, and the Wald test was used to assess statistical significance. Forest plots were used to visualize the results of the analysis of the effects of the interactions of LCR with other variables on the overall survival of the participants. Calibration curves were used to visualize the differences between the predicted and observed probabilities. The accuracy of predictions made using the LCR risk score was assessed using the area under the ROC curve. All the tests performed were two-sided and *P*<0.05 was considered to represent statistical significance. Statistical analyses were performed using R software version 4.0.2 (R Foundation for Statistical Computing, Vienna, Austria), and the packages ‘survminer’, ‘survival’, ‘rms’,’ timeROC’, ‘forestplot’, ‘ggrisk’, ‘ggplot2’, ‘ggsci’, ‘tableone’, and ‘dplyr’.

## Results

### Participant characteristics and the relationships between LCR and other variables

**Figure 1:**
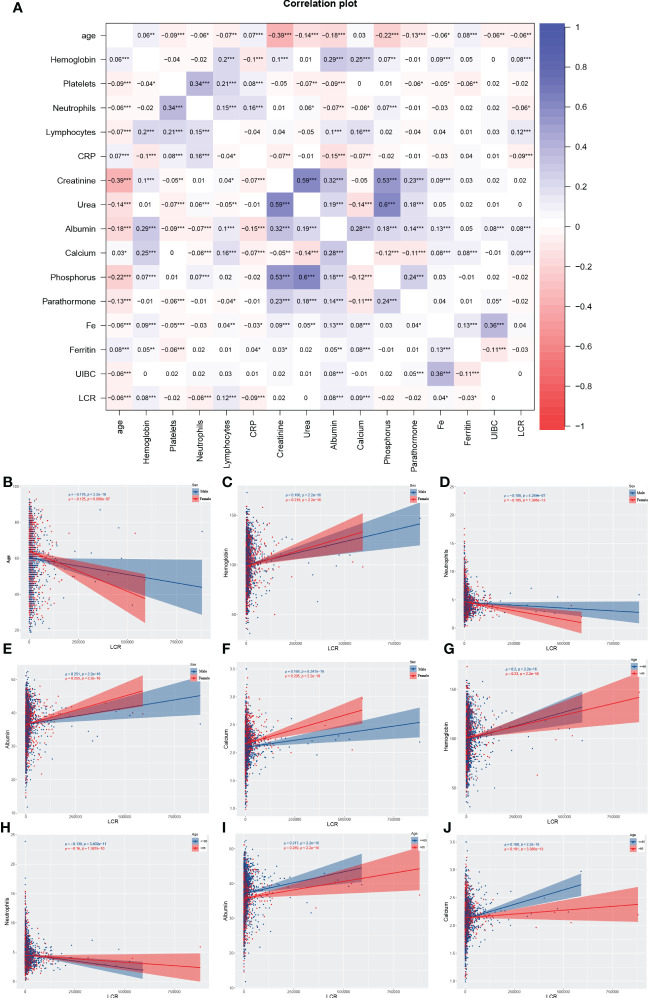
Results of the Spearman analysis of the relationship between LCR and other variables. In the entire cohort **(A)**; in the subgroups of sex with respect to age **(B)**, hemoglobin level **(C)**, neutrophil count **(D)**, serum albumin **(E)**, and serum calcium **(F)**; and in the subgroups according to age with respect to hemoglobin level **(G)**, neutrophil count **(H)**, serum albumin **(I)**, and serum calcium **(J)**. *P<0.05, **P<0.01, ***P<0.001.

From the original total of 6,126 patients, we excluded 2,270 for the reasons listed above, leaving a total of 3,856 participants in the cohort. They had a median age of 62 (53, 73) years and 1,581 (41%) were female. The overall characteristics of the participants and the results of their stratification according to the calculated LCR cut-off value are presented in **Table 1**. In summary, 1,129 deaths were recorded over a median follow-up period of 75.1 months. The overall mortality rate for the participants 60 months after their initial assessment was 38.1% (95% CI 36%–40.1%), resulting in a rate of 93.41 events per 1,000 patient-years. The log LCR was compared among the various sex and age groups comprising the sample. The results showed that young (<65 years old) participants had a significantly higher LCR than older (≥65 years old) participants (*P*<0.001) (**Figure S2**). Moreover, Spearman analysis of the relationships of LCR with various parameters that are clinically relevant for patients undergoing hemodialysis showed significant correlations with age, hemoglobin level, neutrophil count, albumin concentration, and calcium concentration. On the basis of these results, we also conducted a stratified analysis of participants according to their age (≥65 years old *vs*. <65 years old) and sex (female *vs*. male), which showed that hemoglobin level, albumin concentration, and calcium concentration positively correlated with LCR in individuals of differing age and sex, while the neutrophil count negatively correlated (**Figure 1**).

**Table 1:** Demographic and clinical characteristics of the full cohort of patients undergoing hemodialysis and after stratification according to LCR.

Characteristic, n (%) or median (IQR)		Overall n=3856	LCR-low n=1223	LCR-high n=2633		*P* value
Population characteristic Sex						
Female		1581 (41.0)	513 (41.9)	1068 (40.6)		0.437
Male		2275 (59.0)	710 (58.1)	1565 (59.4)		
Age		62.00 (53.00, 73.00)	65.00 (55.50, 74.00)	61.00 (52.00, 71.00)		<0.001
Clinical characteristic, median (IQR)						
Hemoglobin (g/L)		101.00 (86.00, 114.00)	94.00 (80.00, 108.50)	103.00 (89.00, 116.00)		<0.001
Platelets(10^9^/L)		174.00 (138.00, 220.00)	173.00 (135.00, 225.50)	174.00(140.00, 218.00)		0.894
Neutrophils (10^9^/L)		4.25 (3.30, 5.39)	4.53 (3.46, 5.94)	4.14 (3.24, 5.13)		<0.001
Lymphocytes (10^9^/L)		1.18 (0.87, 1.56)	0.91 (0.55, 1.24)	1.29 (0.99, 1.67)		<0.001
CRP (mg/L)		3.34 (1.28, 9.30)	14.34 (8.46, 28.54)	2.00 (0.98, 3.98)		<0.001
Creatinine (umol/L)		703.00 (535.30, 896.00)	664.00 (491.50, 855.50)	723.00 (563.00, 912.00)		<0.001
Urea (mmol/L)		22.09 (17.56, 27.80)	21.10 (16.87, 27.60)	22.56 (17.93, 27.83)		0.001
Albumin (g/L)		37.10 (33.80, 40.10)	35.40 (31.90, 38.60)	38.00 (34.80, 40.70)		<0.001
Calcium (mmol/L)		2.14 (1.99, 2.27)	2.10 (1.95, 2.23)	2.16 (2.02, 2.29)		<0.001
Phosphorus (mmol/L)		1.64 (1.29, 2.04)	1.61 (1.25, 2.00)	1.65 (1.30, 2.06)		0.015
Parathormone (pg/ml)		200.00 (103.20, 346.16)	199.16 (103.95, 359.35)	200.70 (103.10, 340.60)		0.760
Fe (mmol/L)		10.20 (7.60, 13.96)	8.90 (6.50, 12.30)	10.90 (8.17, 14.45)		<0.001
Ferritin (ng/ml)		231.15 (99.95, 456.33)	262.20 (111.00, 507.39)	216.00 (96.90, 433.90)		<0.001
UIBC (umol/L)		41.40 (35.39, 48.90)	39.70 (33.58, 47.50)	42.20 (36.30, 49.20)		<0.001

Data are shown as median (interquartile range) or number (%); IQR, interquartile range; LCR, lymphocyte-to-C reactive protein ratio. The LCR-low group had an LCR <1513.1 and the LCR-high group had an LCR ≥1513.1.

### Relationship of LCR with overall survival

**Figure 2:**
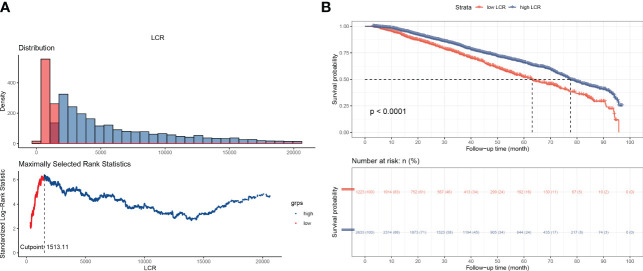
Overall survival of the participants, according to LCR category. **(A)** Outcome-oriented method, determining the optimal cut-off point of LCR to be 1513.1. **(B)** Kaplan-Meier analysis of overall survival, based on the calculated cut-off value.

Potential risk factors for all-cause mortality were identified using univariate and multivariate Cox proportional hazards regression analyses (**Table 2**). Univariate analysis revealed that age, platelet count, lymphocyte count, CRP, creatinine, urea, albumin, phosphorus, ferritin, and LCR were associated with the overall survival of the participants. The multivariate analysis revealed that age, platelet count, albumin, phosphorus, and LCR were independent predictors of mortality. Then, according to the results of the multivariate analysis, we evaluated the prognostic value of combinations of LCR with the other independent prognostic factors, and the 1, 3, 5, and 7-year calibration curves showed that these combinations would be extremely useful for the prediction of survival (**Figure S3**). The cut-off point of LCR determined by using Kaplan-Meier curves was 1513.1 (**Figure 2A**), of which hemodialysis patients with an LCR <1513.1 were found to be associated with higher mortality.

**Table 2:** Results of the univariate and multivariate Cox regression analyses to identify factors associated with overall survival.

Characteristic	No. of Event/Median (IQR)	Univariate analysis		Multivariate analysis			
		HR (95% CI)	*P-*value	HR (95% CI)	*P* value		
Sex							
Female	1581 (41.0)	Ref		Ref		
Male	2275 (59.0)	0.93 (0.82-1.04)	0.192	1.08 (0.95-1.21)	0.244	
Age	62.00 (53.00, 73.00)	1.05 (1.04-1.05)	<0.001	1.04(1.04-1.05)	<0.001	
Hemoglobin (g/L)	101.00 (86.00, 114.00)	1.00 (0.99-1.01)	0.257			
Platelets(10^9^/L)	174.00 (138.00, 220.00)	0.99 (0.99-1.00)	0.007	0.99 (0.99-1.00)	0.026	
Neutrophils (10^9^/L)	4.25 (3.30, 5.39)	1.00 (0.96-1.03)	0.843			
Lymphocytes (10^9^/L)	1.18 (0.87, 1.56)	0.88 (0.79-0.97)	0.014			
CRP (mg/L)	3.34 (1.28, 9.30)	1.00 (1.00-1.01)	0.011			
Creatinine (umol/L)	703.00 (535.30, 896.00)	0.99 (0.99-1.00)	<0.001	0.99 (0.99-1.00)	0.059	
Urea (mmol/L)	22.09 (17.56, 27.80)	0.98 (0.97-0.99)	<0.001	0.99 (0.98-1.99)	0.130	
Albumin (g/L)	37.10 (33.80, 40.10)	0.95 (0.94-0.97)	<0.001	0.97 (0.96-0.98)	<0.001	
Calcium (mmol/L)	2.14 (1.99, 2.27)	1.13 (0.90-1.42)	0.288			
Phosphorus (mmol/L)	1.64 (1.29, 2.04)	0.83 (0.75-0.91)	<0.001	1.16 (1.02-1.32)	0.025	
Parathormone (pg/ml)	200.00 (103.20, 346.16)	1.00 (0.99-1.01)	0.602			
Fe (mmol/L)	10.20 (7.60, 13.96)	1.00 (0.99-1.01)	0.249			
Ferritin (ng/ml)	231.15 (99.95, 456.33)	1.00 (1.00-1.01)	0.003	1.00 (1.00-1.01)	0.070	
UIBC (umol/L)	41.40 (35.39, 48.90)	0.99 (0.99-1.01)	0.279			
LCR	314.50 (105.46, 906.42)	0.99 (0.99-1.00)	<0.001	1.00 (0.99-1.00)	0.004	

Data are shown as median (interquartile range) or number (%); IQR, interquartile range; LCR, lymphocyte-to-C reactive protein ratio.

**Figure 3:**
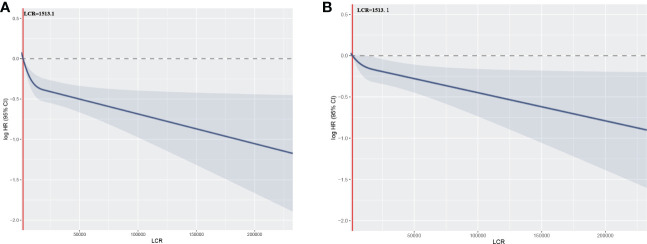
Relationships between LCR, as a continuous variable, and the hazard ratio for overall survival. Restricted cubic splines (RCSs) were used. **(A)** Unadjusted restricted cubic spline for LCR; **(B)** RCS adjusted for sex, age, hemoglobin level, platelet count, neutrophil count, creatinine, urea, albumin, calcium, parathormone, phosphorus, Fe, ferritin, and UIBC.

To further evaluate the relationship between LCR and mortality in patients undergoing hemodialysis, four categories of LCR were defined. First, as a continuous variable, the restricted cubic spline plot showed that LCR had an L-shaped dose-response relationship with the risk of all-cause mortality risk in the participants (**Figure 3**). Second, the Cox regression models of the relationship between LCR and OS showed that LCR positively correlated with prognosis (HR 0.85 per SD increase, 95% CI 0.75–0.95, *P*=0.006) after adjustment for sex, age, hemoglobin level, platelet count, neutrophil count, creatinine, urea, albumin, calcium, parathormone, phosphorus, Fe, ferritin, and unsaturated iron-binding capacity (UIBC) (**Table 3**). Third, using the cut-off value calculated for LCR, the participants were divided into two groups: a low-LCR group and a high-LCR group. Compared with the low-LCR group, participants in the high-LCR group had a consistently better prognosis (HR 0.75, 95% CI 0.66–0.85, *P*<0.001) after adjustment for the variables listed above. Finally, the participants were divided into quartiles according to their LCR; and compared with the first quartile (Q1, <1,054.3), the second (1,054.3–3,144.9), third (3,144.9–9,068.6), and fourth quartiles (≥9,068.6) all had a better prognosis (*P* for trend <0.05). After adjustment for the potential confounding factors, the HRs for all-cause mortality were 0.78 (95% CI 0.66–0.92, *P*=0.003), 0.73 (95% CI 0.62–0.87, *P*<0.001), and 0.76 (95% CI 0.64–0.91, *P*=0.003) for the second, third, and fourth quartiles, respectively.

**Table 3:** Relationships between LCR and overall survival in the participants.

LCR	Crude model		Model A		Model B	
	HR (95% CI)	*P* value	HR (95% CI)	*P* value	HR (95% CI)	*P* value
As continuous (per SD)	0.79 (0.70-0.90)	<0.001	0.84 (0.74-0.95)	0.005	0.85 (0.75-0.95)	0.006
By LCR cut-off						
Low (<1513.1)	Ref		Ref		Ref	
High (≥1513.1)	0.66 (0.58-0.74)	<0.001	0.71 (0.63-0.80)	<0.001	0.75 (0.66-0.85)	<0.001
Interquartile						
Q1 (<1054.3)	Ref		Ref		Ref	
Q2 (1054.3-3144.9)	0.74 (0.63-0.87)	<0.001	0.74 (0.63-0.87)	<0.001	0.78 (0.66-0.92)	0.003
Q3 (3144.9-9068.6)	0.66 (0.56-0.77)	<0.001	0.70 (0.60-0.83)	<0.001	0.73 (0.62-0.87)	<0.001
Q4 (≥9068.6)	0.60 (0.51-0.71)	<0.001	0.70 (0.59-0.83)	<0.001	0.76 (0.64-0.91)	0.003
P for trend		<0.001		<0.001		0.003

The results of the crude model are shown. Model A was adjusted for sex and age; and Model B was adjusted for sex, age, hemoglobin level, platelet count, neutrophil count, creatinine, urea, albumin, calcium, parathormone, phosphorus, Fe, ferritin, and UIBC. CI, confidence interval; HR, hazard ratio; Q, quartile; LCR, lymphocyte-to-C reactive protein ratio.

### Demographics and disease characteristics of the participants after stratification according to LCR

According to the cut-off point calculated for LCR, the 3,856 participants were divided into two groups: a low-LCR group (LCR <1513.1, n=1,223) and a high-LCR group (LCR ≥1513.1, n=2633). The Kaplan-Meier curves and log-rank test results revealed that the high-LCR group had a better prognosis than the low-LCR group (**Figure 2B**). **Table 1** presents a comparison of the demographics and clinical characteristics of the low- and high-LCR groups. Briefly, the participants in the low-LCR group were older; and had lower hemoglobin, higher neutrophil counts, lower lymphocyte counts, higher CRP, lower creatinine, lower urea, lower albumin, lower calcium, lower phosphorus, lower Fe, higher ferritin, and lower UIBC than those in the high-LCR group.

### Results of the stratification analysis

**Figure 4:**
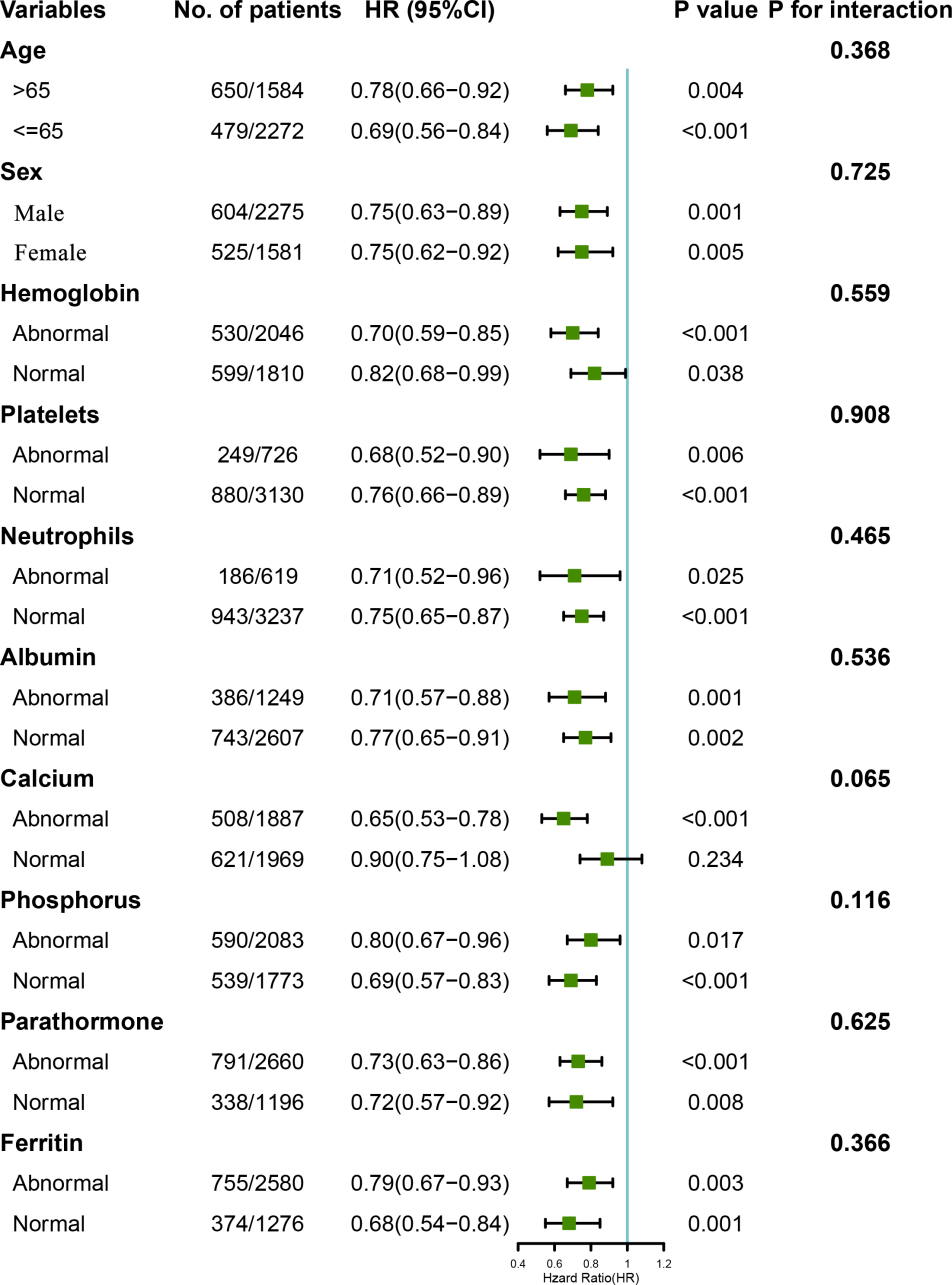
Relationships between LCR category, based on the calculated cut-off value, and the hazard ratios for overall survival in the subgroups. The model was adjusted for sex, age, hemoglobin level, platelet count, neutrophil count, creatinine, urea, albumin, calcium, parathormone, phosphorus, Fe, ferritin, and UIBC. The normal ranges for each parameter were as follows: hemoglobin 100–130 g/L, platelet count 125–350×10^9^/L, neutrophil count 1.8–6.3×10^9^/L, albumin >35 g/L, calcium 2.1–2.52 mmol/L, parathormone 150–300 pg/μl, phosphorus 1.13–1.78 mmol/L, and ferritin 200–500 ng/ml.

Stratified analyses were conducted to evaluate the relationships between LCR and the HR for overall mortality in various subgroups (**Figure 4** and **Table S1**). Overall, high LCR was consistently associated with a lower risk of death in the participants, irrespective of their subgrouping. However, although the same trend was also present in participants with a normal calcium concentration, it was not statistically significant (*P*=0.234). Moreover, an analysis was performed to explore the interactions between high LCR and the other variables, but this showed no associations between high LCR and low overall risk of mortality in the participants (*P* for interaction >0.05 in all instances). In addition, the LCR and covariates were then cross-classified to better understand the effects of each variable (**Table S2**). This analysis showed that an abnormal status with regard to any of a number of variables and a low LCR had an additive effect to increase the risk of mortality. Kaplan-Meier curves also showed that combinations of low LCR and abnormalities in other variables had a deleterious effect on mortality. Specifically, participants with an LCR of <1513.1 had the worst survival rate when they were ≥ 65 years old and had an abnormal albumin concentration (**Figure S4**).

### Results of the sensitivity analysis and internal validation

To validate the finding that LCR is a useful predictor of mortality in patients undergoing hemodialysis, a sensitivity analysis and internal validation were performed to assess the robustness of the results (**Table 4**). An analysis performed after excluding the participants who died within 6 months of the first assessment showed that LCR remained an independent predictor of mortality (adjusted HR 0.85, 95% CI 0.75–0.96, *P*=0.007 for a high LCR per SD). Subsequently, the full cohort was randomly assigned at a 7:3 ratio to validation cohort A (n=2,681) or validation cohort B (n=1,175) using computer-generated random numbers (**Table S3**). Similar results were obtained for cohort A (adjusted HR 0.87, 95% CI 0.78–0.98, *P*=0.026 for high LCR per SD) and cohort B (adjusted HR 0.69, 95% CI 0.49–0.97, *P*=0.034 for high LCR per SD). Moreover, Kaplan-Meier curves showed that participants with a high LCR had a better prognosis when in either validation cohort A or B (**Figures S5A, B**).

**Table 4:** Results of the sensitivity analysis and internal validation of the relationship between LCR and overall survival.

LCR Sensitive analysis	Crude model		Model A		Model B	
	HR (95% CI)	*P* value	HR (95% CI)	*P* value	HR (95% CI)	*P* value
Excluding patients dying within 6 months						
As continuous (per SD)	0.80 (0.70-0.91)	<0.001	0.84 (0.75-0.95)	0.006	0.85 (0.75-0.96)	0.007
By LCR cut-off						
Low (<1513.1)	Ref		Ref		Ref	
High (≥1513.1)	0.66 (0.58,0.75)	<0.001	0.70 (0.62-0.80)	<0.001	0.74 (0.65-0.85)	<0.001
Interquartile						
Q1 (<1054.3)	Ref		Ref		Ref	
Q2 (1054.3-3144.9)	0.73 (0.62-0.86)	<0.001	0.73 (0.62-0.86)	<0.001	0.77 (0.65-0.91)	0.002
Q3 (3144.9-9068.6)	0.68 (0.58-0.81)	<0.001	0.73 (0.62-0.86)	<0.001	0.76 (0.64-0.80)	0.002
Q4 (≥9068.6)	0.61 (0.52-0.72)	<0.001	0.71 (0.60-0.84)	<0.001	0.77 (0.64-0.92)	0.004
P for trend		<0.001		<0.001		0.007
Validation cohort A						
As continuous (per SD)	0.83 (0.73-0.94)	0.04	0.87 (0.77-0.98)	0.024	0.87 (0.78-0.98)	0.026
By LCR cut-off						
Low (<1513.1)	Ref		Ref		Ref	
High (≥1513.1)	0.7 (0.6-0.81)	<0.001	0.74 (0.64-0.86)	<0.001	0.76 (0.65-0.88)	0.008
Interquartile						
Q1 (<1054.3)	Ref		Ref		Ref	
Q2 (1054.3-3144.9)	0.75 (0.61-0.9)	0.003	0.75 (0.61-0.91)	0.003	0.76 (0.62-0.93)	0.008
Q3 (3144.9-9068.6)	0.7 (0.58-0.85)	<0.001	0.74 (0.61-0.9)	0.002	0.75 (0.61-0.92)	0.005
Q4 (≥9068.6)	0.68 (0.56-0.83)	<0.001	0.78 (0.64-0.95)	0.014	0.81 (0.66-0.99)	0.045
P for trend		<0.001		0.018		0.061
Validation cohort B						
As continuous (per SD)	0.63 (0.45-0.89)	0.009	0.67 (0.47-0.95)	0.025	0.69 (0.49-0.97)	0.034
By LCR cut-off						
Low (<1513.1)	Ref		Ref		Ref	
High (≥1513.1)	0.57 (0.46-0.72)	<0.001	0.64 (0.51-0.81)	<0.001	0.7 (0.55-0.88)	0.003
Interquartile						
Q1 (<1054.3)	Ref		Ref		Ref	
Q2 (1054.3-3144.9)	0.74 (0.56-0.98)	0.037	0.73 (0.55-0.97)	0.028	0.85 (0.63-1.13)	0.263
Q3 (3144.9-9068.6)	0.56 (0.41-0.76)	<0.001	0.62 (0.45-0.83)	0.002	0.65 (0.47-0.89)	0.008
Q4 (≥9068.6)	0.46 (0.34-0.63)	<0.001	0.56 (0.41-0.77)	<0.001	0.66 (0.47-0.94)	0.019
P for trend		<0.001		<0.001		0.006

The results of the crude model are shown. Model A was adjusted for sex and age; and Model B was adjusted for sex, age, hemoglobin level, platelet count, neutrophil count, creatinine, urea, albumin, calcium, parathormone, phosphorus, Fe, ferritin, and UIBC. CI, confidence interval; HR, hazard ratio; Q, quartile; LCR, lymphocyte-to-C reactive protein ratio.

### Construction of a risk score model

**Figure 5:**
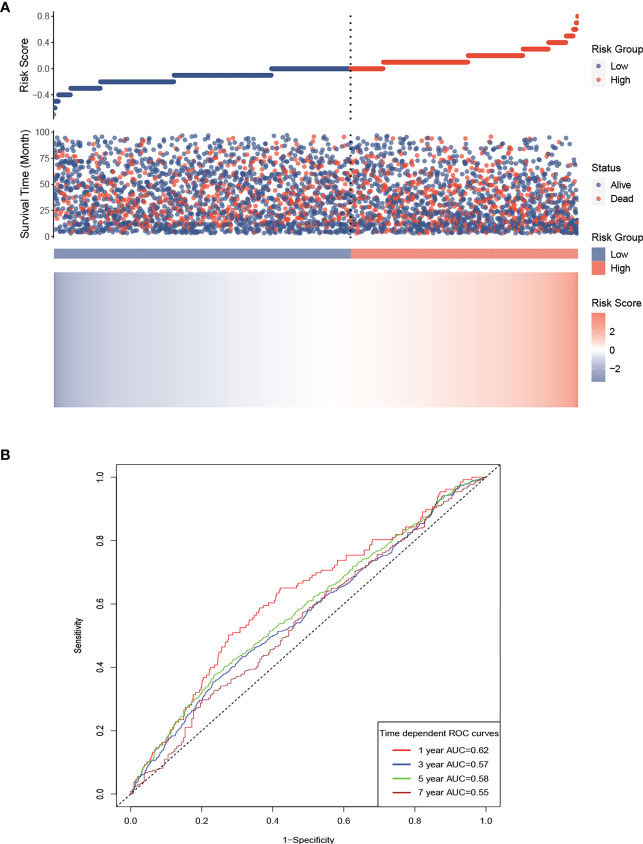
Risk score model for the participants, based on LCR. Prognostic risk score model for the participants, based on LCR. **(A)** High LCR is associated with a lower risk score and a better prognosis. **(B)** AUCs of 62.0, 56.5, 58.3, and 54.9 were calculated for 1, 3, 5, and 7 years, respectively. AUC, area under the curve.

LCR values were naturally log-transformed to make it easier to assess the prognosis of patients undergoing hemodialysis, and then a risk score model was constructed using the β regression risk coefficient derived from the multivariate Cox regression model and the log LCR. The formula for the risk score was determined to be −0.1669 × log LCR. Using the calculated risk scores, a risk plot heatmap and a time-dependent ROC curve were created. The results, shown in **Figure 5A**, indicate that a high LCR is associated with a low risk score and a better prognosis. **Figure 5B** shows AUCs of 62.0, 56.5, 58.3, and 54.9 for 1, 3, 5, and 7 years, respectively.

## Discussion

In the present study, we have demonstrated the prognostic utility of LCR for patients undergoing hemodialysis in a number of ways. First, we have shown that the baseline LCR is an independent predictor and has an L-shaped relationship with all-cause mortality in these patients. Second, we have calculated a specific cut-off value of LCR for use with patients undergoing maintenance hemodialysis and demonstrated that baseline LCR values above this level are associated with better survival. Third, we have constructed a prognostic risk model using LCR that is an excellent predictor of prognosis. Finally, we have performed sensitivity and internal validation analyses in which we obtained similar results.

Inflammation is a physiological response to various deleterious stimuli under normal conditions and represents the first stage of healing. Cohen et al. measured the circulating pro-inflammatory cytokine (IL-1, IL-6, and TNF-α) concentrations of 231 patients undergoing hemodialysis and found that those with high concentrations had shorter survival times than the others, which demonstrates that biomarkers of the severity of inflammation are independent and reliable prognostic indicators in such patients (24). In the context of chronic kidney disease, and especially in patients undergoing hemodialysis, unmanaged and sustained systemic inflammation caused by uremic toxins, oxidative stress, fluid overload, and/or artificial materials have been identified to be critical in patients with cardiovascular disease, malnutrition, and anemia, and are associated with high mortality (25), and this is consistent with the results of the Spearman analysis in the present study, which showed that hemoglobin level, albumin, and calcium positively correlate with LCR in individuals of various ages and sexes.

The concentrations of specific pro-inflammatory cytokines, such as IL-1, IL-6, MMP-9, and TNF-α, can often not be measured routinely, and such assays are often not affordable for use with patients with hemodialysis, rendering them of limited use in clinical practice. Identifying useful and readily measurable biomarkers of inflammation and identifying patients at high risk would therefore be extremely valuable to permit early intervention (lifestyle, medication, or the modification of dialysis). A number of markers of inflammation, such as NLR, PLR, PNI, and GPS, have been reported to be a useful means of evaluating the prognosis of patients with hemodialysis, but none have become established as gold standards for the evaluation of the inflammatory status of patients. In the present study, we studied the utility of LCR, a newly developed marker of inflammation.

Lymphocytes play an important role in the cytotoxic immune response, and the loss of circulating lymphocytes is associated with a loss of immunological specificity, resulting in higher all-cause mortality in older individuals (26). A previous study of the relationship between white blood cell count and mortality in 44,114 patients undergoing hemodialysis showed that a high lymphocyte count is associated with a lower risk of mortality (HR 0.86, 95% CI 0.83–0.89, *P*<0.001) (27). CRP has served as a useful marker of infection and tissue inflammation for several decades, and has been shown to be regulated by the pro-inflammatory cytokines TNF-α and IL-6 in an *in vitro* study (28). In addition, linear risk relationships of CRP with coronary heart disease, stroke, and mortality in the healthy population have been found. In a large international multi-center cohort study of patients undergoing hemodialysis, CRP was found to be a better predictor of mortality 1 year later than circulating ferritin concentration and white blood cell count (29). Consistent with the results of a previous study, both the lymphocyte count and the C-reactive protein concentration were found to be predictors of mortality in the present study. LCR was calculated using the lymphocyte count and the CRP concentration, which is less variable and a better predictor than either lymphocyte count or CRP alone, and was first studied and shown to have prognostic value with respect to colorectal cancer by Okugawa et al. in 2020, after various combinations of pro-inflammatory marker concentrations in preoperative blood samples, including neutrophil count, lymphocyte count, CRP concentration, albumin concentration, and platelet count, were evaluated (18).

In recent years, the use of a high LCR as a predictor of overall survival has been validated in patients with stomach cancer, hepatocellular carcinoma, bladder cancer, or rectal cancer (19–23). The results of the present study are consistent with the published literature because high LCR was found to be consistently associated with better overall survival in patients undergoing hemodialysis and in the various subgroups. To the best of our knowledge, this is the first study to demonstrate the value of LCR for the prediction of overall survival in patients undergoing hemodialysis. Thus, LCR represents a valid means of assessing the prognosis of patients at the initiation of hemodialysis. In future studies, the prognostic utility of LCR, PLR, NLR, and PNI in such patients would be worth comparing.

In the present study cohort, hemodialysis patients with an LCR <1513.1 was found to be associated with higher mortality, which differs from that previously reported for use in patients with cancer. The cut-off value calculated for colorectal cancer was 6,000 and that for intrahepatic cholangiocarcinoma was 7,873.1 (18, 30), both of which are higher than that calculated in the present study. This may be explained by the LCR cut-off value being associated with differing levels of inflammation or comorbidities under the various disease conditions. The stratified analysis performed in the present study shows that LCR has prognostic value when used alongside various clinical parameters, low LCR patients with an abnormal clinical parameter always have an additive effect in increasing the risk of mortality, but that there are no significant interactions between them. We also found that there is a positive correlation between LCR and circulating albumin concentration, which reflects nutritional status relatively well. Moreover, when the participants were categorized according to the cut-off value for LCR, those in the low-LCR group had low creatinine, urea, and albumin concentrations. This may be explained by the presence of malnutrition-inflammation-cachexia syndrome in these patients with chronic kidney disease, but multiple mechanisms are likely to be involved. Increases in the concentrations of pro-inflammatory cytokines can induce anorexia, which is accompanied by chronic fatigue and the breakdown of muscle proteins, ultimately leading to a reduction in nutrient intake, greater resting energy expenditure, and muscle atrophy (31, 32). Patients with low LCR are more likely to develop malnutrition-inflammation-cachexia syndrome, involving low circulating creatinine, urea, and albumin concentrations.

Thus, considering the results of our study that hemodialysis patients with low LCR levels are associated with a poor outcome and sustained inflammatory status, early intervention in improving patient’s outcome could be done in the below three aspects. First, building a healthy lifestyle with a balanced diet, regular physical exercise and quitting smoking (33, 34). Second, choosing the right medication which has proved to have a positive effect on inflammation when dealing with the complications of CKD or other comorbidities, such as angiotensin-converting enzyme inhibitors in the treatment of hypertension and sevelamer in the treatment of hyperphosphatemia (35, 36). Third, formulating appropriate dialysis strategies, such as increasing the frequency of dialysis, the application of hemodiafiltration (HDF) or longer dialysis sessions (37).

To the best of our knowledge, this is one of the largest studies to evaluate the relationship between markers of inflammation and the survival of patients undergoing hemodialysis, and the only study to assess whether LCR is independently associated with their survival. However, the present study had several limitations. First, other conventional markers of systemic inflammation, such as the platelet-to-lymphocyte ratio and the neutrophil-to-lymphocyte ratio, were not analyzed in this study, and additional parameters, such as IL-6 and TGF-β, should be included in more comprehensive evaluations. Second, whether variability in serial LCRs over the course of hemodialysis in individual patients is associated with clinical outcomes should be evaluated, to determine whether LCR values calculated at time-points other than baseline also have prognostic value. Third, the present study was a multi-center retrospective study, and there would have been some unidentified confounders that could have contributed to bias in the data obtained. Further well-designed prospective trials are necessary to circumvent this limitation. Finally, external validation of the findings should be performed using large samples in multiple geographical regions, to permit the generalization of the findings to all patients undergoing hemodialysis.

In conclusion, baseline LCR is an independent prognostic marker in patients who are undergoing hemodialysis, with high LCR being associated with superior outcomes. This implies that the calculation of LCR may be a useful means of improving patient prognosis and identifying the appropriate timing for interventions in routine clinical practice.

## Data availability statement

The raw data supporting the conclusions of this article will be made available by the authors, without undue reservation.

## Ethics statement

The studies involving human participants were reviewed and approved by Human Ethics Committees of Beijing Friendship Hospital. Written informed consent for participation was not required for this study in accordance with the national legislation and the institutional requirements.

## Author contributions

XC and WG conceived the study. WG provided the data. XC and WG analyzed the results and wrote the manuscript. XC, WG, ZD, WL, and HH analyzed the results and reviewed the manuscript. ZD, WL, and HH had full access to all the data in the study and take responsibility for the integrity of the data and the accuracy of the data analysis. All the authors contributed to the article and approved the submitted version.

## Supplementary material

Supplementary Figure 1 Flow chart for the study.

Supplementary Figure 2 Distribution of log LCR in individuals undergoing hemodialysis in each sex and age group.

Supplementary Figure 3 Calibration curves for the use of LCR in combination with sex, age, platelet count, albumin, and phosphorus.

Supplementary Figure 4 Kaplan-Meier curves for the overall survival of the participants. The participants were divided into groups according to the LCR cut-off and covariates. *P*-values were derived using the log-rank test.

Supplementary Figure 5 Overall survival of the participants, categorized according to the LCR cut-off value, in the validation cohorts. Kaplan-Meier curves of overall survival for groups based on the calculated cut-off value of LCR. **(A)** Validation cohort A; **(B)** Validation cohort B.
