# Supplementary material for: Lymphocyte-to-C reactive protein ratio as novel inflammatory marker for predicting outcomes in hemodialysis patients: A multicenter observational study
Source: Front Immunol. 2023 Mar 2;14:1101222. doi: 10.3389/fimmu.2023.1101222 (PMC10017876; doi:10.3389/fimmu.2023.1101222)
Supplement: Supplementary file 1 [file Table_1.docx]

Table S1. Associations between LCR (stratified by cut-offs 1513.1) and hazard ratios for overall survival in various subgroups.

| Variables | No. of patients | HR (95%CI) | *P* value | *P* for interaction |  |
| --- | --- | --- | --- | --- | --- |
| Age |  |  |  | 0.368 |  |
| >65 | 650/1584 | 0.78(0.66-0.92) | 0.004 |  |  |
| <=65 | 479/2272 | 0.69(0.56-0.84) | <0.001 |  |  |
| Sex |  |  |  | 0.725 |  |
| Male | 604/2275 | 0.75(0.63-0.89) | 0.001 |  |  |
| Female | 525/1581 | 0.75(0.62-0.92) | 0.005 |  |  |
| Hemoglobin | |  |  | 0.559 |  |
| Abnormal | 530/2046 | 0.70(0.59-0.85) | <0.001 |  |  |
| Normal | 599/1810 | 0.82(0.68-0.99) | 0.038 |  |  |
| Platelets |  |  |  | 0.908 |  |
| Abnormal | 249/726 | 0.68(0.52-0.90) | 0.007 |  |  |
| Normal | 880/3130 | 0.76(0.66-0.89) | <0.001 |  |  |
| Neutrophils | |  |  | 0.465 |  |
| Abnormal | 186/619 | 0.71(0.52-0.96) | 0.025 |  |  |
| Normal | 943/3237 | 0.75(0.65-0.87) | <0.001 |  |  |
| Albumin |  |  |  | 0.536 |  |
| Abnormal | 386/1249 | 0.71(0.57-0.88) | 0.001 |  |  |
| Normal | 743/2607 | 0.77(0.65-0.91) | 0.002 |  |  |
| Calcium |  |  |  | 0.065 |  |
| Abnormal | 508/1887 | 0.65(0.53-0.78) | <0.001 |  |  |
| Normal | 621/1969 | 0.90(0.75-1.08) | 0.234 |  |  |
| Phosphorus | |  |  | 0.116 |  |
| Abnormal | 590/2083 | 0.80(0.67-0.96) | 0.017 |  |  |
| Normal | 539/1773 | 0.69(0.57-0.83) | <0.001 |  |  |
| Parathormone | |  |  | 0.625 |  |
| Abnormal | 791/2660 | 0.73(0.63-0.86) | <0.001 |  |  |
| Normal | 338/1196 | 0.72(0.57-0.92) | 0.008 |  |  |
| Ferritin |  |  |  | 0.366 |  |
| Abnormal | 755/2580 | 0.79(0.67-0.93) | 0.003 |  |  |
| Normal | 374/1276 | 0.68(0.54-0.84) | 0.001 |  |  |

Except the stratifying variable, the model is adjusted by sex, age, hemoglobin level, platelets count, neutrophils count, creatinine level, urea level, albumin level, calcium level, parathormone level, phosphorus level, Fe level, ferritin level and UIBC level.

Table S2. Multivariate models for LCR and covariates

|  |  | **Model a** | | **Model b** | |
| --- | --- | --- | --- | --- | --- |
| **Characteristic** | | **HR (95% CI)** | ***P* value** | **HR (95% CI)** | ***P* value** |
| **LCR** | **Age** |  |  |  |  |
| Low | ≥65 | 3.50(2.95-4.14) | <0.001 | 2.99(2.50-3.57) | <0.001 |
| Low | <65 | 1.51(1.25-1.83) | <0.001 | 1.45(1.19-1.77) | <0.001 |
| High | ≥65 | 2.52(2.18-2.92) | <0.001 | 2.31(1.98-2.69) | <0.001 |
| High | <65 | Ref | Ref | Ref | Ref |
| **LCR** | **Sex** |  |  |  |  |
| Low | Male | 1.48(1.25-1.76) | <0.001 | 1.48(1.24-1.77) | <0.001 |
| Low | Female | 1.44(1.21-1.73) | <0.001 | 1.37(1.14-1.65) | 0.001 |
| High | Male | 1.08(0.93-1.25) | 0.320 | 1.13(0.97-1.31) | 0.114 |
| High | Female | Ref | Ref | Ref | Ref |
| **LCR** | **Hemoglobin** | |  |  |  |
| Low | Abnormal | 1.37(1.16-1.61) | <0.001 | 1.22(1.02-1.46) | 0.033 |
| Low | Normal | 1.36(1.14-1.60) | 0.001 | 1.28(1.07-1.52) | 0.007 |
| High | Abnormal | 0.93(0.81-1.08) | 0.366 | 0.89(0.76-1.03) | 0.116 |
| High | Normal | Ref | Ref | Ref | Ref |
| **LCR** | **Platelets** |  |  |  |  |
| Low | Abnormal | 1.74(1.40-2.16) | <0.001 | 1.66(1.33-2.07) | <0.001 |
| Low | Normal | 1.39(1.21-1.60) | <0.001 | 1.33(1.14-1.53) | <0.001 |
| High | Abnormal | 1.23(1.03-1.47) | 0.023 | 1.23(1.03-1.48) | 0.024 |
| High | Normal | Ref | Ref | Ref | Ref |
| **LCR** | **Neutrophils** | |  |  |  |
| Low | Abnormal | 1.52(1.23-1.88) | <0.001 | 1.53(1.23-1.91) | <0.001 |
| Low | Normal | 1.38(1.2-1.59) | <0.001 | 1.3(1.13-1.50) | <0.001 |
| High | Abnormal | 1.05(0.84-1.32) | 0.648 | 1.04(0.83-1.31) | 0.711 |
| High | Normal | Ref | Ref | Ref | Ref |
| **LCR** | **Albumin** |  |  |  |  |
| Low | Abnormal | 1.70(1.43-2.01) | <0.001 | 1.81(1.51-2.17) | <0.001 |
| Low | Normal | 1.32(1.13-1.55) | 0.001 | 1.32(1.12-1.56) | 0.001 |
| High | Abnormal | 1.21(1.03-1.43) | 0.023 | 1.26(1.06-1.50) | 0.008 |
| High | Normal | Ref | Ref | Ref | Ref |
| **LCR** | **Calcium** |  |  |  |  |
| Low | Abnormal | 1.42(1.20-1.68) | <0.001 | 1.23(1.02-1.47) | 0.028 |
| Low | Normal | 1.27(1.07-1.51) | 0.006 | 1.18(0.99-1.40) | 0.072 |
| High | Abnormal | 0.90(0.78-1.04) | 0.164 | 0.82(0.71-0.96) | 0.013 |
| High | Normal | Ref | Ref | Ref | Ref |
| **LCR** | **Phosphorus** | |  |  |  |
| Low | Abnormal | 1.34(1.12-1.59) | 0.001 | 1.42(1.20-1.68) | <0.001 |
| Low | Normal | 1.56(1.31-1.86) | <0.001 | 1.27(1.07-1.51) | 0.006 |
| High | Abnormal | 1.04(0.90-1.21) | 0.564 | 0.90(0.78-1.04) | 0.164 |
| High | Normal | Ref | Ref | Ref | Ref |
| **LCR** | **Parathormone** | |  |  |  |
| Low | Abnormal | 1.40(1.17-1.67) | <0.001 | 1.34(1.12-1.59) | 0.001 |
| Low | Normal | 1.33(1.06-1.66) | 0.014 | 1.56(1.31-1.86) | <0.001 |
| High | Abnormal | 0.97(0.83-1.14) | 0.714 | 1.04(0.90-1.21) | 0.564 |
| High | Normal | Ref | Ref | Ref | Ref |
| **LCR** | **Ferritin** |  |  |  |  |
| Low | Abnormal | 1.48(1.25-1.77) | <0.001 | 1.40(1.17-1.67) | <0.001 |
| Low | Normal | 1.49(1.20-1.84) | <0.001 | 1.33(1.06-1.66) | 0.014 |
| High | Abnormal | 1.09(0.93-1.27) | 0.293 | 0.97(0.83-1.14) | 0.714 |
| High | Normal | Ref | Ref | Ref | Ref |

Model a: adjusted by sex and age;

Model b: adjusted by sex, age, hemoglobin level, platelets count, neutrophils count, creatinine level, urea level, albumin level, calcium level, parathormone level, phosphorus level, Fe level, ferritin level and UIBC level.

Table S3 Demographic and clinical characteristics of validation cohort

| Characteristic, n (%) or median (IQR) | Cohort A n=2681 | Cohort B n=1175 | *P* value |
| --- | --- | --- | --- |
| Population characteristic |  |  |  |
| Sex |  |  |  |
| Female | 1089 (40.6) | 492 (41.9) | 0.489 |
| Male | 1592 (59.4) | 683 (58.1) |  |
| Age | 62.00 (53.00, 72.00) | 63.00 (53.00, 73.00) | 0.770 |
| Clinical characteristic, median (IQR) |  |  |  |
| Hemoglobin (g/L) | 101.00 (86.00, 114.00) | 101.00 (87.00, 114.00) | 0.865 |
| Platelets(10^9^/L) | 176.00 (138.00, 221.00) | 171.00 (138.00, 217.00) | 0.048 |
| Neutrophils (10^9^/L) | 4.25 (3.30, 5.41) | 4.26 (3.25, 5.31) | 0.621 |
| Lymphocytes (10^9^/L) | 1.18 (0.87, 1.56) | 1.17 (0.86, 1.55) | 0.479 |
| CRP (mg/L) | 3.31 (1.27, 9.30) | 3.38 (1.28, 9.34) | 0.780 |
| Creatinine (umol/L) | 705.00 (542.60, 895.60) | 699.00 (519.35, 897.00) | 0.466 |
| Urea (mmol/L) | 22.12 (17.58, 27.83) | 22.00 (17.56, 27.60) | 0.453 |
| Albumin (g/L) | 37.00 (33.70, 40.00) | 37.30 (33.80, 40.50) | 0.288 |
| Calcium (mmol/L) | 2.14 (1.99, 2.26) | 2.14 (2.00, 2.28) | 0.397 |
| Phosphorus (mmol/L) | 1.63 (1.29, 2.04) | 1.65 (1.29, 2.05) | 0.790 |
| Parathormone (pg/ml) | 198.70 (103.10, 346.00) | 206.60 (103.70, 346.67) | 0.596 |
| Fe (mmol/L) | 10.23 (7.60, 13.80) | 10.20 (7.52, 14.20) | 0.816 |
| Ferritin (ng/ml) | 229.40 (99.81, 453.90) | 237.90 (100.30, 461.05) | 0.517 |
| UIBC (umol/L) | 41.40 (35.40, 49.26) | 41.40 (35.23, 48.00) | 0.240 |
| LCR | 3177.93 (1076.26, 9112.48) | 3030.50 (980.14, 9039.57) | 0.461 |

Data are represented as mean (SD), median (interquartile range) or number (%); IQR, interquartile range.
