# Supplementary figures and images for: Lymphocyte-to-C reactive protein ratio as novel inflammatory marker for predicting outcomes in hemodialysis patients: A multicenter observational study
Source: Front Immunol. 2023 Mar 2;14:1101222. doi: 10.3389/fimmu.2023.1101222 (PMC10017876; doi:10.3389/fimmu.2023.1101222)

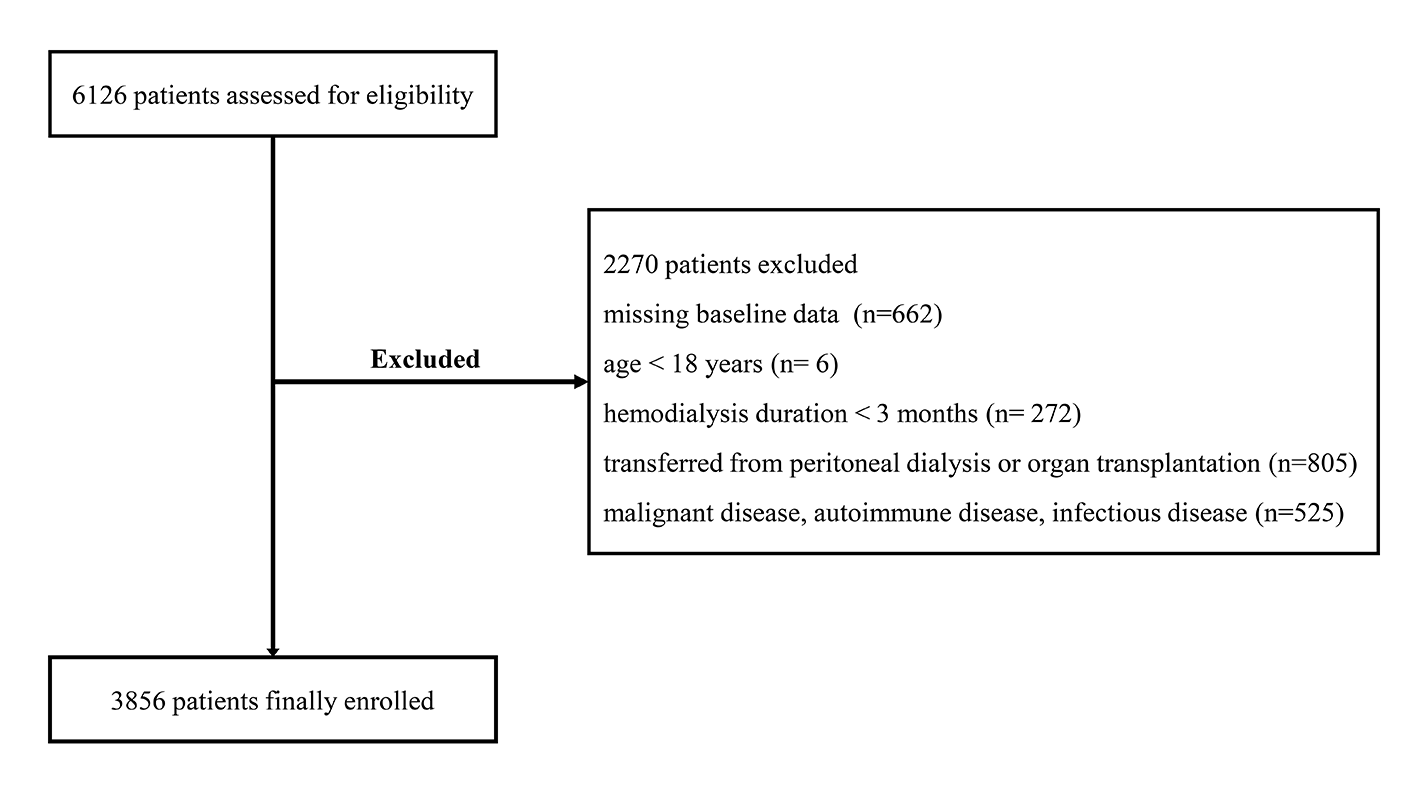

Supplement: Supplementary Figure 1 — Flow chart for the study. [file Image_1.tif]

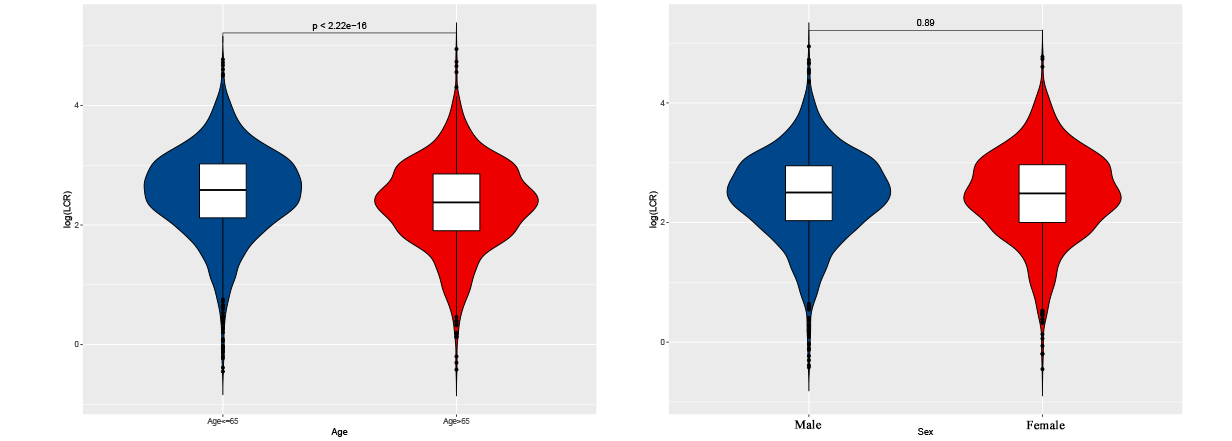

Supplement: Supplementary Figure 2 — Distribution of log LCR in individuals undergoing hemodialysis in each sex and age group. [file Image_2.tif]

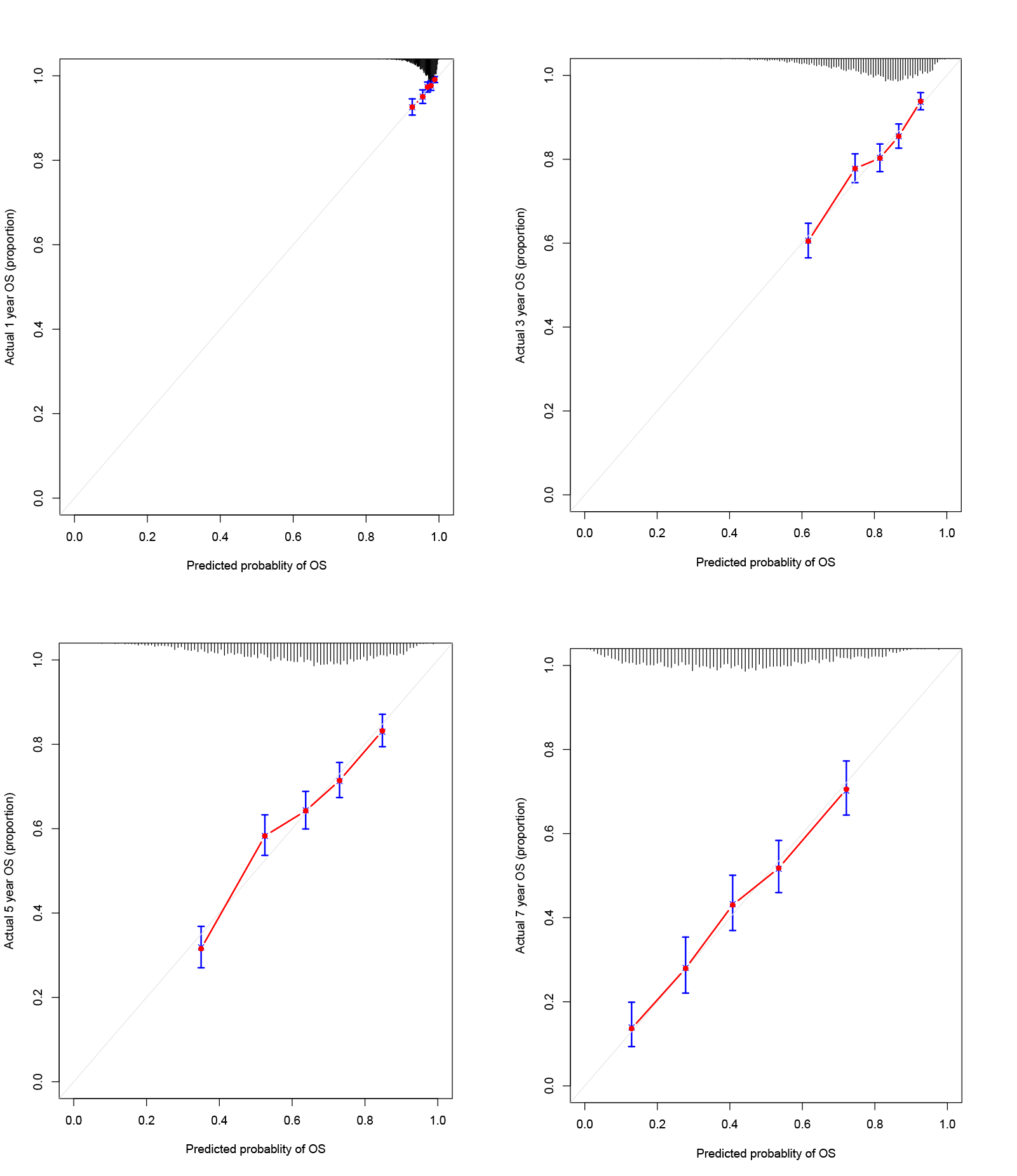

Supplement: Supplementary Figure 3 — Calibration curves for the use of LCR in combination with sex, age, platelet count, albumin, and phosphorus. [file Image_3.tif]

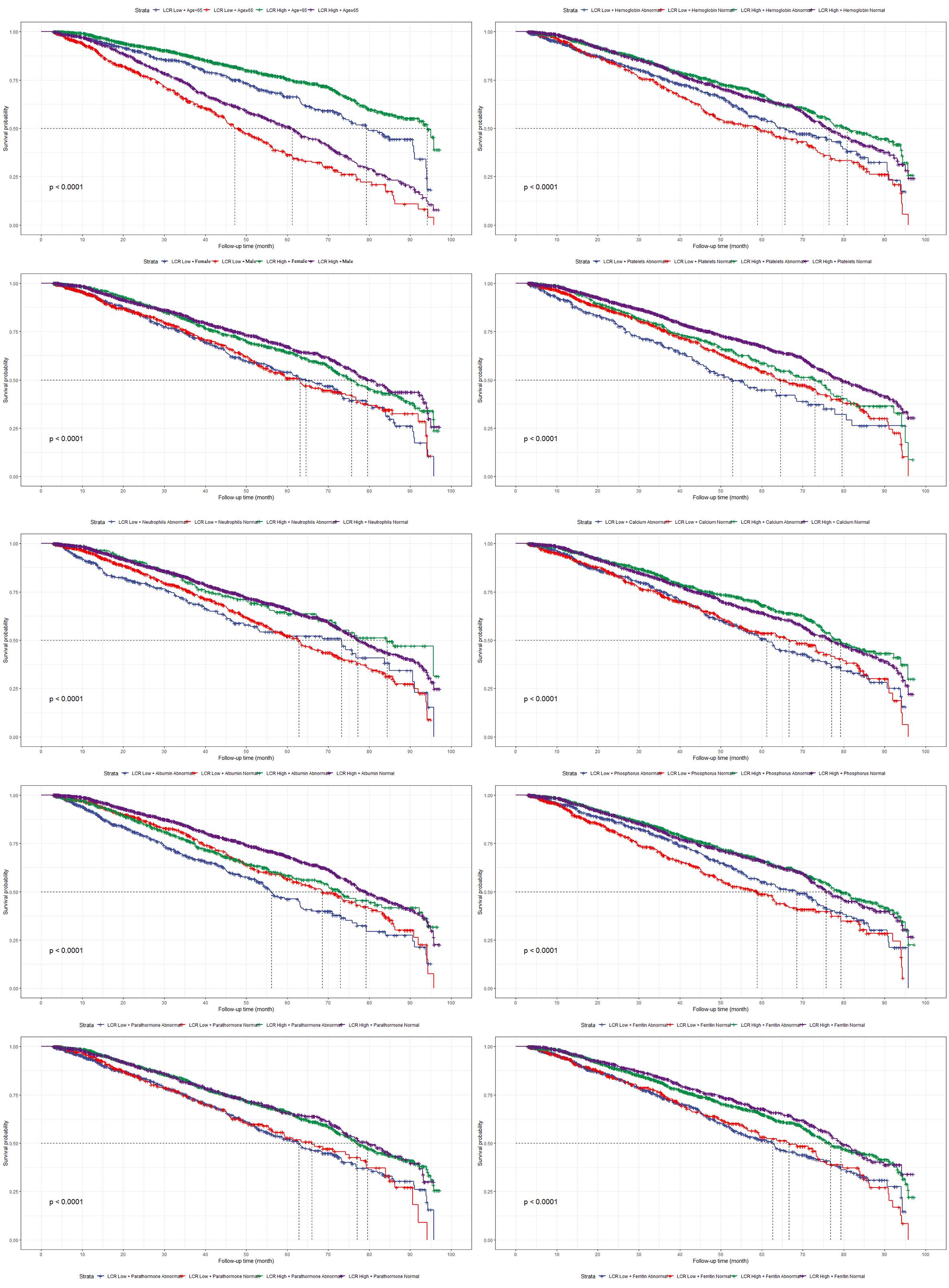

Supplement: Supplementary Figure 4 — Kaplan-Meier curves for the overall survival of the participants. The participants were divided into groups according to the LCR cut-off and covariates. P-values were derived using the log-rank test. [file Image_4.tif]

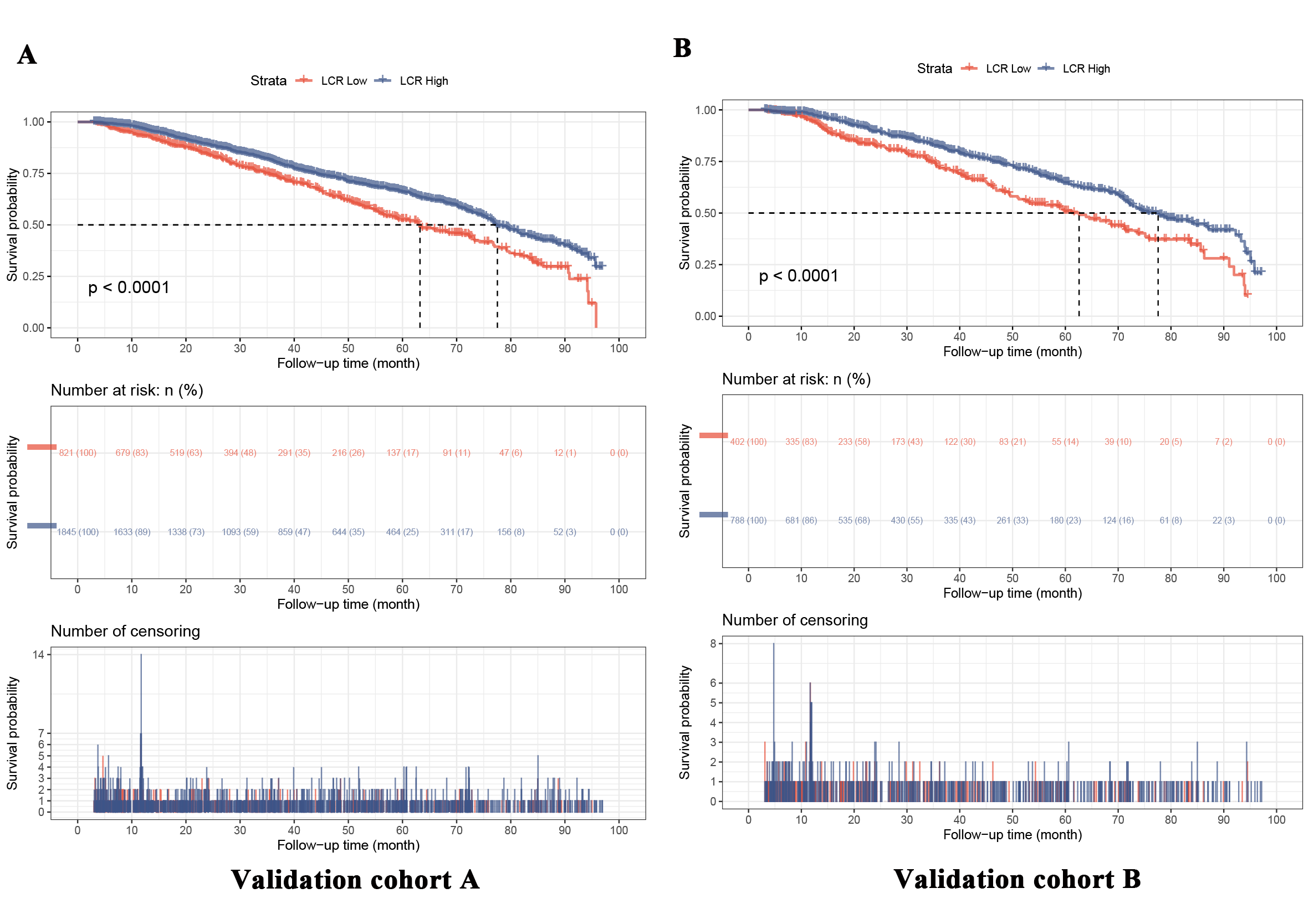

Supplement: Supplementary Figure 5 — Overall survival of the participants, categorized according to the LCR cut-off value, in the validation cohorts. Kaplan-Meier curves of overall survival for groups based on the calculated cut-off value of LCR. (A) Validation cohort A; (B) Validation cohort B. [file Image_5.tif]
